# The effects of short video app-guided loving-kindness meditation on college students’ mindfulness, self-compassion, positive psychological capital, and suicide ideation

**DOI:** 10.1186/s41155-023-00276-w

**Published:** 2023-10-30

**Authors:** Chao Liu, Hao Chen, Ayuan Zhang, XiaoGang Gong, Kan Wu, Chia-Yih Liu, Wen-Ko Chiou

**Affiliations:** 1 School of Journalism and Communication, Hua Qiao University, Xiamen, 361021 China; 2 Business Analytics Research Center, Chang Gung University, Taoyuan, 33302 Taiwan; 3 School of Film Television & Communication, Xiamen University of Technology, Xiamen, China; 4 Teachers College, Beijing Union University, Beijing, 100101 China; 5 College of Special Education, Beijing, 100101 China; 6 Department of Psychiatry, Chang Gung Memorial Hospital, Taoyuan, Taiwan; 7 Department of Industrial Engineering and Management, Ming Chi University of Technology, New Taipei, Taiwan; 8 Department of Industrial Design, Chang Gung University, Taoyuan, Taiwan

**Keywords:** Short video app, Loving-kindness meditation, Mindfulness, Self-compassion, Positive psychological capital, Suicide ideation

## Abstract

**Objective:**

The study investigated the effects of a short video app guided loving-kindness meditation (LKM) on college students’ mindfulness, self-compassion, positive psychological capital, and suicide ideation. The purpose of the study is to investigate the intervention effect of LKM training on suicidal ideation among college students with the help of the short video application and to provide an empirical basis for the exploration of early suicide intervention strategies for college students.

**Methods:**

We recruited 80 college students from a university in China. The final 74 eligible participants were divided into two groups: app use group (*n* = 37) and the control group (*n* = 37). The app group accepted an 8-week app use interference, while the control group underwent no interference. We measured four major variable factors (mindfulness, self-compassion, positive psychological capital, and suicide ideation) before and after the app use intervention.

**Results:**

In the app group, self-compassion and positive psychological capital were significantly higher, and suicide ideation was significantly lower than the control group. In the control group, there were no noticeable differences in any of the four variables between the pre-test and post-test.

**Conclusions:**

Our findings demonstrate that the short video app guided LKM may help to improve self-compassion, and positive psychological capital, and reduce suicide ideation. The finding of the short video app-guided LKM’s effect extends our understanding of the integrative effects of positive psychology and digital media on the reduction of suicide ideation.

## Introduction

Based on the data of the World Health Organization, more than 700,000 individuals die of suicide each year worldwide. Suicide has become the fourth main cause of death among people aged 15–29, especially among college students (Liu et al., 2022f). Suicidal ideation is an early psychological activity of suicide. It refers to death, suicide, and serious self-harm in an individual’s thoughts or thoughts, including thoughts about plans, steps, and results of suicide (Zortea et al., 2020). Compared with traditional media, the Internet and social networking sites that are spread described that suicide and suicide knowledge provides more opportunities, according to a study of Chinese students, suicidal ideation and suicide-related highly relevant social media use behavior, including suicide information, reviews, and forward to commit suicide, or talk about suicide-related content (Liu et al., 2022a, 2022b, 2022c, 2022d, 2022e, 2022f). Although the vast majority of suicidal ideations do not lead to actual suicidal actions, suicidal ideation is still considered a necessary condition for suicide and attempted suicide. Therefore, researchers explore the causes of suicidal ideation among college students, to reduce and eliminate suicidal ideation among college students and prevent suicidal behavior (Liao et al., 2022).

Suicidal ideation is closely related to all kinds of risk factors, from the point of suicide to escape and suicide interpersonal theory; an individual’s experience and regulation of negative emotions such as suicidal ideation, for example, stressful events, mood, emotions, and ideas, change which is highly correlated with suicidal ideation; a clinical study manifested that over 80% of all suicides were attacked by mental illness, usually depression, substance abuse, or impulse control disorders (Díaz-Oliván et al., 2021). Based on the six steps of the suicide process described in the theoretical model of self-avoidance, compassion can prevent suicide in six steps: (1) an event or state does not meet one’s expectations and standards; (2) inappropriate internal attribution; (3) feeling incompetent and unliked; (4) negative emotions; (5) cognitive decline, focusing only on immediate activities and feelings; (6) long-term cognition makes death a means to escape bad emotions and painful self-consciousness (Baumeister, 1990). A compassionate attitude can help individuals accept themselves, perceive social support, and regulate negative emotions. In recent years, positive psychology has attracted widespread attention. The nature of positive psychology emphasizes that the development of psychology should not only prevent and treat psychological problems but also pay more attention to the positive role of cultivating and constructing excellent qualities (Seligman, 2019). The suicide buffer hypothesis holds that in addition to the promotion of negative factors, there are also various positive psychological factors that protect suicide.

Positive psychological capital, which includes elements like hope, optimism, self-efficacy, and resilience, plays a crucial role in mitigating suicidal ideation. Positive psychological capital enhances an individual’s psychological resilience. When faced with life’s stresses and setbacks, those with a high level of positive psychological capital are more likely to persevere, seek solutions to problems, and refrain from choosing suicide as an option (Liu et al., 2022a, 2022b, 2022c, 2022d, 2022e, 2022f). Their optimistic outlook and sense of hope can serve as powerful motivators to overcome adversity. Positive psychological capital contributes to improved emotional regulation. Optimistic and self-assured individuals are better equipped to effectively manage negative emotions, reducing emotional distress and the risk of suicidal ideation. They can employ positive thinking and emotional regulation strategies to cope with difficult situations (Liu et al., 2019). Positive psychological capital is closely linked to social support and interpersonal relationships. This psychological state helps in establishing healthier and more stable social networks, which can offer assistance and support when individuals need it most. This is crucial in reducing feelings of loneliness and the risk of suicidal thoughts (Chiou et al., 2021). Positive psychological capital provides a hopeful perspective. For those who feel hopeless and helpless, positive psychological capital offers a way to view the future more positively, instilling the belief that life can improve. This sense of hope can act as a robust shield against suicidal ideation (Chiou et al., 2022). In conclusion, positive psychological capital enhances psychological resilience, emotional regulation, social support, and hopefulness, all of which are vital in reducing the risk of suicidal ideation. Therefore, fostering and promoting positive psychological capital is crucial for maintaining mental health and preventing suicide (Liu et al., 2020a, 2020b, 2020c).

In terms of the intervention and treatment of suicidal ideation, the practical application of mindfulness meditation in the field of psychology has accumulated some achievements (Wielgosz et al., 2019). As the research on mindfulness becomes increasingly saturated, more and more researchers begin to turn to another practice related to mindfulness meditation, namely loving-kindness meditation (LKM) (Condon & Makransky, 2020).

LKM, a method of work that concentrates on blessing oneself and others in the mind, has the effect of generating positive emotions and fostering positive attitudes (Chen et al., 2021a, 2021b; Liu et al., 2020a, 2020b, 2020c, 2022a, 2022b, 2022c, 2022d, 2022e, 2022f). As for the function and effect of LKM, several intervention strategies based on LKM have been developed in the academic circle, mainly focusing on two aspects positive attitude and positive emotion. From the perspective of positive psychological capital, optimism, hope, resilience, and self-efficacy, as important psychological traits, all reflect a positive psychological ability or emotional attitude (Sirotina & Shchebetenko, 2020). A large number of studies have proved that LKM, through blessing oneself or others, generates endogenous love and kindness, increases positive emotional states such as satisfaction, joy, and optimism (Zeng et al., 2019), and has an important effect on cultivating pro-social attitudes (Koopmann-Holm et al., 2021).

LKM has significant effects on mindfulness and self-compassion. Self-compassion is the capacity to convey understanding as well as kindness to oneself, especially to bless oneself when feeling frustrated in life (Neff & Germer, 2022; Tóth-Király & Neff, 2021). Roca found that both MBSR (mindfulness-based stress reduction) programs and LKM training increased mindfulness, decentralization, body awareness, and self-compassion (Roca et al., 2021a, 2021b; Roca et al., 2021a, 2021b). Li et al. developed a 4-week online course on LKM for 65 participants and found that LKM improved daily self-compassion and reduced perceived stress and emotional exhaustion (Li et al., 2021). Meanwhile, LKM has an indirect impact on psychological health through self-compassion and the existence of vita meaning as well as the reduction of experiential avoidance (Yela et al., 2020).

LKM plays a role in the therapy of pressure, inquietude, depression, marital conflict, and post-traumatic stress disorder as well as other specific problems involved in interpersonal relationships (Zeng et al., 2015). LKM has the potential to reduce depressive symptoms in a wide population. Symptoms of depression here include sadness, lack of pleasure, changes in eating and sleeping, feelings of meaninglessness, and even suicidal intentions (Graser & Stangier, 2018). Neuroendocrine studies have shown that LKM can reduce subjective distress and immune responses to stress, with significant effects on mental distress (i.e., pressure, inquietude, and depression), and accommodate cognitive processes (i.e., regurgitate and thought suppression) (Schlosser et al., 2022).

## Research gap

Over the past decade, the use of the Internet to treat mental health problems, including the prevention of suicidal ideation and behavior, has become increasingly common (Bailey et al., 2020). In the treatment of suicide ideation, traditional psychological intervention on suicide usually adopts the prevention of negative factors to intervene, but with the rise of positive psychology theory, researchers began to pay attention to the prevention of suicide ideation through protective factors. As a high-risk group for suicidal ideation, college students have always been an important concern in various fields. In order to take targeted suicide prevention measures and carry out suicide ideation research and other practical work, it is necessary to explore specific forms of suicide ideation intervention for college students. However, there is a shortage of evidence-based methods for taking precautions against suicide ideation among college students, which cannot provide practical guidance for suicide prevention and intervention.

At present, domestic and foreign studies on suicide intervention mainly focus on the control of risk factors, there is lack of attention to the protective factors of positive psychological orientation, and there are only a few studies through mindfulness training (including mindful cognitive therapy, mindful stress therapy, mindful groups, etc.) to help college students reduce suicide tendency (Raj et al., 2019; Smit & Stavrulaki, 2021). Relevant studies indicate that although LKM can improve positive emotions and reduce depressive symptoms, no studies have directly measured the intervention effect on suicidal ideation (Silhan, 2022; Totzeck et al., 2020). Therefore, the purpose of the research is to investigate the intervention effect of LKM training on suicidal ideation among college students with the help of the short video application and to provide an empirical basis for the exploration of early suicide intervention strategies for college students. The study aims to verify whether short video application guided LKM can enhance college students’ mindfulness, self-compassion, and positive psychological capital and reduce suicidal ideation among college students. On account of the above thesis and basis, we present the following hypotheses:

- Hypothesis 1 (H1): A short video app-guided LKM interposition can significantly lessen the level of suicide ideation of the participants.
- Hypothesis 2 (H2): A short video app-guided LKM interposition can significantly enhance the level of mindfulness of the participants.
- Hypothesis 3 (H3): A short video app-guided LKM interposition can significantly enhance the level of positive psychological capital of the participants.
- Hypothesis 4 (H4): A short video app-guided LKM interposition can significantly enhance the level of self-compassion of the participants.

## Method

### Participants

A total of 5043 freshmen from a university in China were investigated for suicidal ideation through an online questionnaire. The initial screening questionnaire was carried out using the 1–5 questions of the Beck Scale for Suicidal Ideation (BSSI). The counselors of each department were organized by psychological professionals. Before the test, unified and standardized guidelines were used to explain the purpose and methods of the test as well as the principle of confidentiality. The questionnaires were automatically numbered by the system, and the invalid questionnaires were screened out and imported into the SPSS23 software. The database was established, and the missing values were checked and processed. A total of 4588 qualified and valid initial screening samples were recovered, and the recovery rate of valid initial screening samples was 90.98%.

Through the overall data entry, combined with questionnaire analysis and preliminary screening criteria, 387 college students met the criteria of suicidal ideation (non-zero score of BSSI question 4 or 5) in the initial screening, accounting for 8.44% of the total valid initial screening sample. The above 387 college students were invited to conduct detailed interviews by telephone appointment. The interview was conducted by psychiatrists, to screen whether any students were suffering from mental illness and needed medical treatment and intervention.

After the interview, the researchers gave online LKM training to all the college students who met the inclusion criteria, introducing the concept of LKM and the purpose, process, and form of online LKM. A total of 80 students were recruited according to the principle of voluntary participation, and 74 eligible subjects participated in the whole process. The participants were split into two groups randomly: the LKM practice group guided by an online short video (37 subjects) and the control group (37 subjects). There was no remarkable discrepancy in demographic elements such as age composition and gender ratio between the two groups (Table 1).

**Table 1:** Demographic characteristics of participants

Characteristic	Total	App group	Control group
Age (SD)	17.73 (1.59)	18.08 (1.61)	17.38 (1.52)
Male (%)	29 (39.2%).	13 (35.1%).	16 (43.2%).
Female (%)	45 (60.8%).	24 (64.9%).	21 (56.8%).

### Instruments

The Mindfulness Attention Awareness Scale (MAAS) was used to survey individual features of mindfulness, compiled by Brown and Ryan (Brown & Ryan, 2003) and revised by Deng et al. (Deng et al., 2012): single-dimensional structure, 15 items, 6 points, with “1” to “6” meaning “almost always” to “almost never”; the higher the score, the more mindful. Among many tools for surveying feature mindfulness, MAAS is the most widely used (Park et al., 2013). A range of research has manifested that MAAS has good credibility and validity in people with distinctive cultural and mindfulness meditation experiences (MacKillop & Anderson, 2007). In this research, Cronbach’s alpha was 0.85 (pre-test) and 0.89 (post-test).

#### Psychological capital

The Psychological Capital Questionnaire (PCQ) was developed by Harms et al. (Harms et al., 2017). The scale contains four dimensions, including self-efficacy, prospect, sanguine, and resilience, with six questions for each dimension and a total of 24 questions. The Chinese version of PCQ was translated by Yan et al. (Yan et al., 2020). The credibility and validity of the PCQ questionnaire have been confirmed in previous studies (Wang et al., 2012; Zhang et al., 2014). In this research, Cronbach’s alpha was 0.87 (pre-test) and 0.91 (post-test).

#### Self-compassion scale (RSCS-C)

The original scale was compiled by Raes et al. (Raes et al., 2011), and the Chinese version interpreted by Meng et al. was adopted in this study (Meng et al., 2019). The internal coherence coefficient of the scale, namely Cronbach’s alpha, was 0.68. There are 12 items in total, including 6 dimensions of self-friendliness, mindfulness, universal humanity, self-criticism, sense of isolation, and over-immersion. For each item, very inconformity = 1, inconformity = 2, neutral = 3, conformity = 4, and very conformity = 5 are selected to score. The higher the total score, the higher the self-compassion level of the subjects. A great deal of research has indicated that RSCS-C has good credibility and validity (Chu et al., 2018; Neff et al., 2019). In this research, Cronbach’s alpha was 0.92 (pre-test) and 0.89 (post-test).

The Beck Scale of Suicidal Ideation (BSSI), developed by Beck et al. (Beck et al., 1979), measures the intensity, duration, and specificity of suicidal plans and desires. The Chinese version contains the following: 0 = “I do not have any suicidal thoughts,” 1 = “I have suicidal thoughts but can’t carry them out,” 2 = “I want to suicide and may suicide”; the total score ranges from 0 to 38, with higher scores indicating greater severity of suicidal ideation. The first five BSSI questions can be used to identify suicidal ideation (Beck & Steer, 1991), where questions 4 and 5 reveal suicidal intent or ideation. If item 4 (positive suicide wish) and item 5 (negative suicide wish) were both rated as 0, the patient had no suicidal ideation and was asked to skip the remaining 14 questions. Only those who scored greater than zero on items 4 or 5 are considered suicidal ideators. BSSI showed sufficient internal consistency (*α* = 0.89) (Beck et al., 1979) as well as a significant correlation with issues of effectiveness and self-harm (Seidman et al., 2022). Numerous studies have shown that BSSI has good credibility and validity (Huang et al., 2022; X. Liu et al., 2020a, 2020b, 2020c). In this research, Cronbach’s alpha was 0.86 (pre-test) and 0.88 (post-test).

### Short video application intervention of LKM

The LKM short video app automatically collects compliance data, including the date, time, and name of the participant viewing, as determined by the researcher. We will send these compliance data to the researchers every 2 weeks for the duration of the study. Taking advantage of this information, the researchers calculated and estimated the total effective time of each participant using the short video application of LKM during the 8-week intervention period (Chen et al., 2022, 2021a, 2021b; Liu et al., 2022a, 2022b, 2022c, 2022d, 2022e, 2022f; Liu et al., 2022a, 2022b, 2022c, 2022d, 2022e, 2022f).

The materials for the short video production were adapted from prior research. The production process involved the creation and design of cartoon characters using a drawing software (Sai). Subsequently, an animation software (Flash) was utilized to craft interactive animations in SWF format, boasting a resolution of 1920 × 1080 pixels. Following this, voice actors were enlisted to record narration materials. The animation is available for a 5-min viewing duration, with the option for continuous looping. Subsequently, the animation was uploaded to a short video application to facilitate monitoring and assessment of each user’s daily viewing and meditation practice time. Leveraging this data, the researchers conducted calculations and assessments to determine the effectiveness of the 8-week intervention period. These meditation intervention materials were designed to offer online LKM training to participants, primarily using animated guidance to instruct participants in the practice of LKM.

The short video application of LKM consisted of animation-guided LKM to conduct the online LKM interposition training. Participants were asked to do the following: (1) put themselves in a state of comfort, sometimes adding pleasurable stimulation; (2) envision the subject being blessed compassionately; (3) with the object in four ways: (a) wish that he/she has no enemies, (b) wish that he/she has no pain, (c) wish that he/she has no sickness, and (d) hope that he/she can have his/her felicity. About the choice of the blessing object, objects generally vary according to the degree of the practitioner’s good deeds and follow the principle from easy to difficult. The general order is as follows: (a) wish happiness to yourself; (b) wish happiness to your loved one; (c) wish happiness to those who are neutral, those you neither like nor hate; (d) wish happiness to those you hate; (e) wish happiness to yourself, your loved ones, the neutral, and the hated simultaneously and equally; (f) wish happiness to all or all living beings. When practitioners are good at evoking compassion for one kind of person, this practice can be transferred to more difficult people. Also, in the practice of LKM, it is significant to avert some subjects, particularly for abecedarians. For example, the object of the blessing must not be of the opposite sex and cannot be too close to oneself or have similar interests, dead people, etc., to ensure the purity of the cultivating feeling (Chen et al., 2021a, 2021b; Liu et al., 2021, 2020a, 2020b, 2020c, 2022a, 2022b, 2022c, 2022d, 2022e, 2022f).

### Procedures and design

The LKM short video app helps participants better understand themselves, improve their mental state, and master ways to love themselves and others. This is the recruitment slogan of this study. College students who were interested and qualified to take part in our research supplied their registration information.

Subjects were enrolled as the following: (1) newly enrolled freshmen; (2) have a non-zero score on question 4 or question 5 of the BSSI scale; (3) have signed the informed consent form and indicated voluntary participation; (4) have clear thinking and normal thinking and are able to communicate with language; and (5) have sufficient cognitive and literacy skills, to complete the questionnaire.

The elimination criteria were as follows: (1) have a history of mental illness or disorder; (2) in the last year, those who have taken psychotropic medications; (3) in the last year, those who have received any form of psychological interposition; and (4) have any form of meditation training experience.

**Fig. 1:**
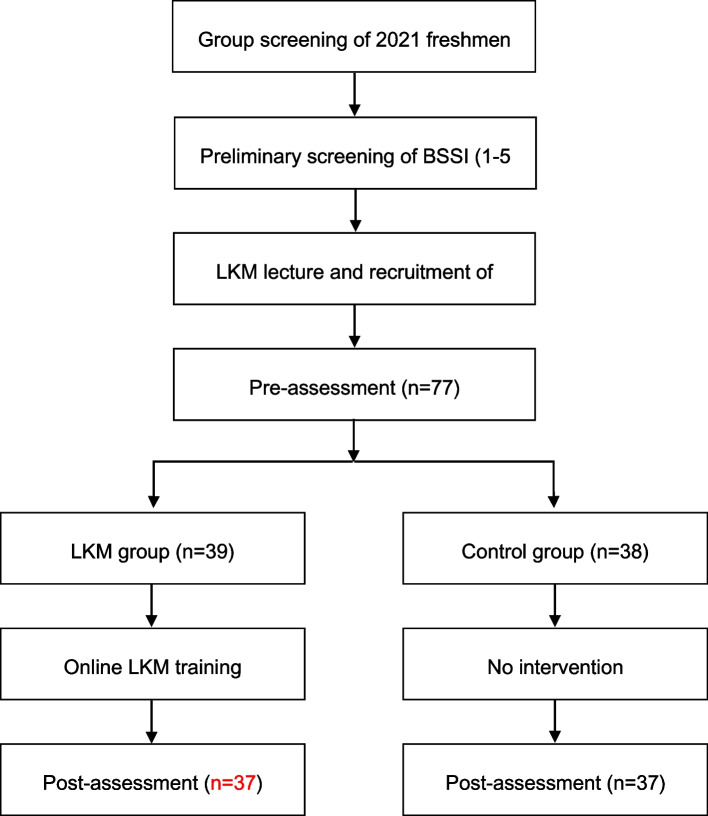
Procedure flow chart

For maintaining engagement and controlling churn, there was a trial subsidy before as well as after the experiment, and each participant received 50 RMB at the beginning of the survey to motivate them. If they are on 8 weeks of daily use of the app intervention with a period of more than 1 h (through the system background app, each user can see the everyday use time), at the end of 8 weeks, the subjects will get RMB 100, and on 8 weeks, if they do not meet the daily app use time, the user will not receive a fee of RMB 100. In the app group, two participants did not meet the requirement of app use duration. The sample size is determined by G*Power. Given *F* = 0.3 for *α* = 0.05, power of 80%, and moderate effect size for correlation between repeated measures, there were 2 sets of 4 measurements. With a recommended sample size of 100 for repeated measures ANOVA, we expected a 20% churn rate from T0 (pretest) to T1 (posttest); thus, we attempted to recruit 80 participants and ended up with 74 valid participants who met the criteria and participated throughout the trial. Participants completed an online approval form through a secure online survey platform, provided demographic information, and completed the following questionnaires (experimental tests): (1) MAAS, (2) PMPS, (3) EPDS, and (4) BSSI. It takes about 20 min to complete the questionnaire. For 8 weeks, the app group received the app intervention three times a week, while the control group did not receive any form of intervention. The experimental group was invited to participate in a 20-min webinar that provided (1) an overview of how to use the mobile app and (2) how to proceed through 8 weeks of online LKM exercises. On Monday following the webinar, all participants started the 8-week LKM exercises. Immediately after the 8-week intervention, post-testing was performed. Participants finished the same questionnaire again and received an extra test fee of 100 RMB at the end of the interposition period (week 8). Following the online funnel reporting procedure, participants were asked if they knew the aim of the research and the topic of the research and if they knew the similar problems in the pretest and posttest. The subjects who were utterly ignorant of the text conditions and innocently practiced the app were recruited, and the funnel report contributed to acquiring a homogeneous swatch in both groups. Any quantity of questionnaires can be recorded anonymously by participants, in addition to which they can opt-out at any phase. The study was confirmed by the Ethics Committee of Chang Gung University (IRB number: 202001014B0D001), and ensuring compliance with the ethics of the Chinese Psychological Society, the protocol was carefully scrutinized. A flowchart of the procedure is shown in Fig. 1.

### Data analysis

The IBM SPSS 23 software was used for statistical analysis, set as the 95% baseline interval and set as the 0.05 significance level. Repeated measures ANOVA was used to compare differences before and after the interposition and between groups, and descriptive statistics was used to depict the distribution of underlying data.

## Results

**Fig. 2:**
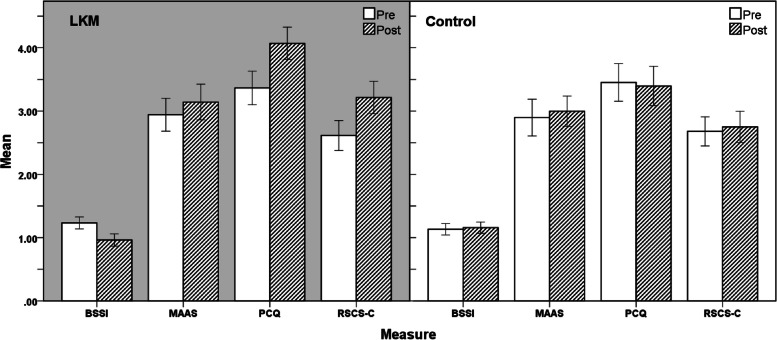
Pairwise comparison between LKM and control group. Errors bars: 95% confidence interval

To contrast the different efforts of online LKM intervention among the two groups, the experiment conducted four repeated measures of ANOVA of 2 (group form: LKM, control) × 2 (time: pre-test, post-test) suicide ideation (BSSI) (Fig. 2), mindfulness (MAAS), positive psychological capital (PCQ), and self-compassion (RSCS-C). The *p*-values of Box’s test, Mauchly’s test, and Levene’s test were all higher than 0.05, demonstrating that these data were appropriate for ANOVAs with repeated measures, and the data followed a normal distribution. Table 2 is the description, and Table 3 is the ANOVA result.

1. Time had a significant main effect on suicidal ideation, with a significant interaction between time and group. The results demonstrated that the online LKM intervention significantly reduced participants’ suicide ideation, and hypothesis 1 was established.
2. There were no significant main effects of time or group on mindfulness and no significant interaction between time and group. The results indicated that the participants’ mindfulness did not significantly improve with the online LKM intervention, so hypothesis 2 was not established.
3. The main effects and interaction effects of time and group on positive psychological were significant. From the results, it can be seen that the positive psychological capital of the participants was significantly increased by the online LKM intervention, and hypothesis 3 was established.
4. The main effect of time on self-compassion was significant, and the interaction between time and group was significant. The results showed that the subjects’ self-compassion was significantly improved by the online LKM intervention, and hypothesis 4 was established.

**Table 2:** Means and standard deviations for each measure of each group, pre- and post-assessment

Group	Measure	Mean (SD)	
		Pre	Post
LKM	BSSI	1.233 (0.284)	0.963 (0.293)
	MAAS	2.942 (0.778)	3.142 (0.847)
	PCQ	3.366 (0.791)	4.070 (0.766)
	RSCS-C	2.615 (0.707)	3.214 (0.769)
Control	BSSI	1.132 (0.272)	1.158 (0.265)
	MAAS	2.897 (0.874)	2.998 (0.722)
	PCQ	3.452 (0.892)	3.396 (0.932)
	RSCS-C	2.679 (0.690)	2.750 (0.739)

**Table 3:** Results of ANOVAs with repeated measures

Measure	Variable	*F*	*p*	*η*^2^
BSSI	Time^**^	7.625	0.007	0.096
	Group	1.000	0.321	0.014
	Time × group^**^	11.188	0.001	0.134
MAAS	Time	1.356	0.248	0.018
	Group	0.483	0.489	0.007
	Time × group	0.148	0.702	0.002
PCQ	Time^*^	4.501	0.037	0.059
	Group^*^	5.513	0.022	0.071
	Time × group^*^	6.211	0.015	0.079
SCS	Time^*^	6.983	0.010	0.088
	Group	3.183	0.079	0.042
	Time × group^*^	4.337	0.041	0.057

^*^p < 0.05

^**^p < 0.01

## Discussion

### LKM short video app can reduce suicidal ideation

The decline of suicidal ideation in the short video application of LKM comes from the following aspects: (1) people with low self-acceptance are more likely to deny themselves, make incorrect internal attributions, and deal with setbacks and dilemmas with negative attitudes. Individuals who participate in LKM are more likely to feel comfortable with themselves by learning more about themselves and identifying their strengths and strengths (Graser & Stangier, 2018). LKM guides college students to know themselves objectively and learn to get along with themselves, and self-spread is to cherish life and cherish the concept of self (Litz & Carney, 2018). (2) Previous research has found that perceptions of social support are inversely related to suicide risk. Perceived social support can reduce suicidal behavior, and the protective effect of social support is not only reflected in the love and kindness from family, friends, and strangers that subjects can feel during the process of LKM (Fredrickson et al., 2019). Effective social support can reduce the risk of suicide by enhancing individual psychological energy and enhancing emotional experience. Suicidal subjects exhibit behavioral and cognitive biases and experience less perceived support within and outside the family (Liu et al., 2022a, 2022b, 2022c, 2022d, 2022e, 2022f). Lower social support makes them less likely to share their inner experiences and release their inner stress, and their risk of suicide increases accordingly (Lin et al., 2020). The social support felt during the practice of LKM can provide individuals with more methods to deal with negative events and reduce suicide risk. (3) Changes in mood and thinking important changes in mood or way of thinking often precede suicide. Intensification of a variety of emotions, including sadness, anger, frustration, anxiety, nervousness, or humiliation, can be a trigger. People with severe psychological distress caused by these emotions often seek relief from suicide (Law & Tucker, 2018). People who commit suicide often develop one or two particular patterns of thought. One is desperation, the firm belief that things will not improve no matter what he does or how circumstances change. Sudden, prolonged feelings of despair are the best cues for suicide (Yan et al., 2020). Through LKM, these experiences and negative emotions can be redirected warmly, and these negative thoughts can also be replaced with compassion (Totzeck et al., 2020).

### LKM short video app can enhance positive psychological capital

The improvement of positive psychological capital in the short video application of LKM comes from the following aspects: (1) the improvement of self-efficacy: self-compassion is a kind of self-compassion and compassion, which is a kind of friendly way to treat oneself in difficulties, as opposed to harsh self-criticism (Engel et al., 2021). Individuals with high levels of self-compassion view their own experiences from a broader perspective, calmly accept the painful experience brought by unfortunate experiences, and do not avoid or overindulge in negative emotions, which is emphasized by the theory of self-compassion (Tóth-Király & Neff, 2021). (2) Improvement of hope and optimism: LKM plays a role in the development of positive emotions. LKM can help college students build the ability to maintain resources and form a positive attitude toward the future. Such college students tend to gain confidence and expectations for the future from positive emotions (Gu et al., 2022). This shows that positive emotions can provide the most critical resources for college students in the early stage, which can help college students establish positive expectations and maintain the growth of resources into a virtuous cycle (Totzeck et al., 2020). At the same time, some researchers believe that interpersonal communication, emotional feedback, and social learning may be the important ways that LKM affects the optimism level of college students (Tellhed et al., 2022). LKM reduced negative emotions, and when the mood was bad, the distraction allowed participants to detach themselves from the constant negative explanations and the resulting negative emotions as quickly as possible (Valim et al., 2019). Maladaptive habits and reaction patterns caused by past experiences cause psychological problems in most subjects. By identifying their advantages and setting up positive expectations, the positive view and interpretation of the event directly lead to the emotional outcome of the event and enhance the hope and optimism of the subjects (Zeng et al., 2019). (3) Improvement of resilience: LKM improves the subjects’ internal and external protective factors, which can significantly improve resilience. LKM promotes positive traits in the individual and can mediate or mitigate the effects of stressful events (Zeng et al., 2017). At the same time, LKM can also improve the participants’ perceived social support. Kindness and connection from family, friends, and colleagues are strong perceived social support, and these external protective factors are factors to improve the impact of crisis (Don et al., 2022).

### LKM short video app can promote self-compassion

There is LKM short video application on the improvement of self-compassion from the following aspects. (1) The higher level of kindness: LKM reduces the subjects of ruminant thinking and avoidance tendency, which can significantly improve yourself; LKM can make fewer subjects addicted to negative events or emotion, to abandon the self-criticism and to treat themselves in a more friendly way (Engel et al., 2021). At the same time, LKM can improve the internalization of individuals by reducing their avoidance tendencies (Litz & Carney, 2018). LKM training was effective in reducing emotional avoidance in the intervention group, who also reported lower levels of anxiety and depression. Therefore, reducing individuals’ avoidance of negative emotions or events is an effective way to produce positive effects of self-compassion (Yela et al., 2022). (2) Improvement of common humanity: LKM improved the emotional regulation strategy using positive cognitive reappraisal. Further studies found that participants who were induced to self-compassion followed by cognitive reappraisal reported lower levels of depression than those who underwent cognitive reappraisal intervention alone, suggesting that LKM can improve the effectiveness of cognitive reappraisal intervention (Dreisoerner et al., 2021). This may be because LKM emphasizes seeing one’s own experience as part of the universal human experience, and this shift in perspective can be beneficial to the individual (Zeng et al., 2015). (3) Reduction of over-identification: LKM can improve subjects’ acceptance level, which can significantly reduce subjects’ over-identification of pain. Some studies have found that LKM can promote personal growth by encouraging participants to embrace their own experiences of regret (Zheng et al., 2022). Another intervention found that training in LKM increased participants’ acceptance of their bodies and reduced their body shame. This increased acceptance of negative experiences is negatively correlated with over-identification with pain (Amy et al., 2020).

### LKM short video app does not promote mindfulness

In this study, participants’ mindfulness did not increase significantly due to the LKM intervention. The process of paying attention to what is going on in the moment is mindfulness, including noticing internal feelings and external stimuli and observing them without making judgments (Kabat-Zinn, 2015). Although mindfulness and LKM share the same element of focus, mindfulness maintains awareness of what is happening inside and outside while focusing on it, while LKM is completely immersed in the process of blessing. They are in a state of selflessness and completely ignore their feelings in the outside world, so they cannot improve their mindfulness (Zeng et al., 2017). In the process of mindfulness meditation, attention is focused on a point of breathing or abdominal fluctuation, and the object of LKM blessing is always changing, so LKM will lead to a certain degree of distraction, which is not conducive to the improvement of mindfulness (Sheldon et al., 2015).

### Limitations and future research limitations of this study

The sample was taken from a university in China. Due to limited research funds, this study could not obtain a larger valid sample size. The small number of respondents may make the findings difficult to further interpret. What is more, since the content of the survey may involve personal privacy, our results relied on self-reported data, which may have included inaccurate responses from participants. (2) As college students with suicidal ideation belong to a specific group, the findings of this study may not apply to other groups, resulting in a low general value of the results. (3) Another limitation is global analysis of positive psychological capital and self-compassion without sub-dimensions. (4) Due to the busy learning nature of college students, it was impossible to put them in a completely closed experimental setting for 8 weeks for the LKM intervention. In addition, subjects must continue to participate in daily study and life during these 8 weeks. The scope of control for this study was smaller than any intervention the subjects experienced. (5) Due to the limitation of research funds, only animation guidance was used in the development process of the short video application of LKM. (6) The short video app itself also has some problems, which may cause students to be too obsessed, or the lack of effective supervision mechanism leads to the worrying quality of some short videos. In the future, a special meditation app can be developed, instead of being attached to short video platforms. In the future, a VR version of the LKM practice guide could be developed. In the future, we will expand the sample collection and add participants from all walks of life; mindfulness, positive psychological capital, compassion, and suicidal ideation are four factors that need more in-depth research. Further discussing the application of LKM short video, it has the same effect on the rest of the sample, with the universality of the test results. Future research will further explore the mechanism by which LKM affects suicidal ideation. This study is primarily focused on highlighting the benefits that LKM can bring to individuals; in future studies, we need to conduct in-depth studies of each factor, integrate various variables, and further explore the relationships among mindfulness, positive psychological capital, self-compassion, and suicidal ideation. From a future perspective, longitudinal studies with repeated follow-up or EEG, fNIRS collection, and analysis in cognitive neuroscience could be added to test the persistence of the efficacy of the short video application of LKM over time.

## Conclusions

Our study showed that self-compassion and positive psychological capital were significantly increased, and suicide ideation was significantly reduced with the help of short video app-guided LKM. The results shed light on the psychological effects of short video app-guided LKM. Our study provides a new value perspective for suicidal ideation intervention from the perspective of positive psychology. These results provide conceptual reference and effective practical significance for expanding and developing the theory of positive psychology. For example, educators and parents can provide more intervention means to promote individual positive psychological capital and self-compassion, to reduce suicidal ideation, which has certain practical significance. With this evidence, programs to intervene with suicidal populations can be designed more precisely. The mechanisms underlying the effects of short video app-guided LKM on self-compassion, positive psychological capital, suicide ideation, and other psychological constructs need to be further elucidated.

## Data Availability

The datasets during the current study are not publicly available due to privacy restrictions but are available from the first author on reasonable request.

## Abbreviations

LKM: Loving-kindness meditation
MAAS: Mindful attention awareness scale
PCQ: Psychological Capital Questionnaire
RSCS-C: Self-compassion scale
BSSI: The Beck Scale of Suicidal Ideation
MBSR: Mindfulness-based stress reduction
